# Concentration Quantification of TiO_2_ Nanoparticles Synthesized by Laser Ablation of a Ti Target in Water

**DOI:** 10.3390/ma15093146

**Published:** 2022-04-26

**Authors:** Damjan Blažeka, Julio Car, Nikša Krstulović

**Affiliations:** Institute of Physics, Bijenička Cesta 46, 10000 Zagreb, Croatia; jcar@ifs.hr (J.C.); niksak@ifs.hr (N.K.)

**Keywords:** nanoparticle concentration, TiO_2_ nanoparticles, laser ablation in liquids, laser synthesis of nanoparticles, Beer–Lambert law, Mie scattering theory

## Abstract

In this work, we present a quantitative method for determining the concentration of metal oxide nanoparticles (NP) synthesized by laser ablation in liquid. The case study was performed with titanium dioxide nanoparticles (TiO_2_ NP), which were synthesized by laser ablation of a Ti target in water. After synthesis, a colloidal solution was analyzed with UV-Vis spectroscopy. At the same time, the craters that remained on the Ti target after ablation were evaluated with an optical microscope to determine the volume of the ablated material. SEM microscopy was used to determine the TiO_2_ NP size distribution. It was found that synthesized TiO_2_ NP followed a Log-Normal diameter distribution with a maximum at about 64 nm. From the volume of ablated material and NP size distribution, under the assumption that most of the ablated material is consumed to form nanoparticles, a concentration of nanoparticles can be determined. The proposed method is verified by comparing the calculated concentrations to the values obtained from the Beer–Lambert law using the Mie scattering theory for the NP cross-section calculation.

## 1. Introduction

Pulsed laser ablation in liquid (PLAL) is a versatile method for the synthesis of colloidal solutions of nanoparticles (NP) [1]. In this method, the laser pulses hit the target immersed in liquid and cause a laser-induced breakdown, followed by the shock wave and plasma plume formation, its expansion and cooling, cavitation bubble formation, and its expansion and collapse [2]. Nanoparticles are produced during the plasma cooling phase in the processes of nucleation and condensation. After the collapse of the cavitation bubble, nanoparticles are released and diffused into the liquid, leading to the formation of a colloidal solution of NP [2]. Because of the liquid environment, the plasma plume is very strongly confined and therefore has a much larger temperature, pressure, and density compared to the ablation in gas [3]. Unlike ablation in gas, there is no need for a closed chamber for NP collection in PLAL. The liquid layer around the plasma plume is also transformed into the plasma phase, leading to a mixture of two plasmas so chemical reactions can take place between them, thus affecting the NP structure [3]. For instance, the ablation of a Ti target in deionized water with a NS laser at a wavelength of 1064 nm results in the synthesis of TiO_2_ NP, as in the present work [4]. 

A PLAL-synthesized colloidal solution of NP usually has large stability due to the presence of a negative charge on the NP surface [5]. The main advantages of PLAL over chemical methods of NP fabrication are the high purity of synthesized nanoparticles, the absence of unwanted residual byproducts in NP colloidal solution, and the possibility of NP synthesis from a large variety of materials [6]. The properties of NP properties, such as morphology, size distribution, and crystal structure, are affected by PLAL parameters such as laser fluence, wavelength, ablation spot size, pulse duration, type of liquid, and presence of surfactants in it. Therefore, there is a large space for modification and engineering of NP properties [7].

The concentration of nanoparticles in colloidal solution is a very important parameter for their application, for example, in biomedical applications [8,9], cancer treatment [10], detectors [11], solar cells, drug delivery, energy storage [12] and photocatalysis [13]. Furthermore, information about NP concentration is important for assessing reproducibility, compliance with regulation, and risk evaluation [14]. Many methods are developed for direct counting or indirect calculation of NP concentration in a colloidal solution. Some of the experimental techniques used for this purpose are [15]: dynamic light scattering (DLS) [16,17], turbidimetry [18,19], optical particle counter [20], NP tracking analysis [17,21,22], single-particle inductively coupled plasma mass spectroscopy (spICPMS) [23], UV-Vis spectroscopy [24,25], resistive-pulse sensing [26], and magnetic NP imaging (MPI) [9]. 

Most of these methods have serious limitations in achieving accurate determinations of NP concentration. For example, the size limitation of NP (in turbidimetry, resistive pulse-sensing, NP tracking analysis), overshadowing the signal of the small nanoparticles (in DLS), or the need for having calibration solution (in DLS and spICMS) [15]. An additional consideration for these methods is the difficulty in sampling, whereby diluting the as-prepared sample over several orders of magnitude is often needed. The main disadvantage of determining concentration from UV-Vis is the need for knowledge of the NP size and refractive indices.

In our previous work [27], a simple model for calculating PLAL-synthesized pure metallic NP concentrations is introduced that considers the volume of an ablated crater and NP size distribution. All disadvantages existing in the previously mentioned methods for concentration calculation do not exist here. The only limitations are those related to the accuracy of crater volume and size distribution determination. This model is successfully tested for Ag NP synthesized by laser ablation of Ag target using Beer–Lambert law. 

In the present work, we show that this model can be adapted for the calculation of the concentration of metal oxide NP synthesized by PLAL of the metallic targets that are very prone to oxidation. The proposed method is tested for TiO_2_ NP synthesized by the laser ablation of a Ti target immersed in water. TiO_2_ NP have stability, corrosion resistance, low reactivity, long agglomeration, and sedimentation time. They have a broad range of industrial and scientific applications, especially photocatalysis [28]. The well-known phases of TiO_2_ are anatase, rutile, and brookite, while amorphous TiO_2_, as one synthesized in this work, is much less investigated. Although amorphous TiO_2_ is characterized by lower photocatalytic performance than one of the crystal phases, it can act as an active component for visible and near-IR light-harvesting, leading to improved photocatalytic activity in this part of the light energy spectrum, so it can also be applied in photodegradation of organic dyes, hydrogen production, and CO_2_ photoreduction [29].

Furthermore, amorphous TiO_2_ has lower toxicity and better corrosion resistance when compared to TiO_2_ in the crystal phase, which can be an advantage in photoprotective and cosmetic applications [30]. It also exhibits excellent antibacterial properties [31], while a special energy band structure and improved charge separation may be advantageous in SERS [29]. Amorphous TiO_2_ NP can be easily transformed to a crystalline phase by heat treatment [29], thus retaining the NP concentration value.

To verify this method, concentrations of TiO_2_ NP calculated from crater volume (*C_V_*) were compared to the concentrations obtained from the UV-Vis photoabsorbance data using Beer–Lambert law and Mie scattering theory (*C_A_*). Beer–Lambert law is usually used for the calculation of the concentration of metal nanoparticles with a well-defined resonant frequency, for example, gold [24,32,33] or silver as in [27]. In the case of TiO_2,_ such calculation is more complicated because it is not plasmonic material. Still, it is a semiconductor that absorbs mainly in the UV range, so it does not possess resonant frequency. Furthermore, TiO_2_ refractive index at some wavelength depends on many parameters such as TiO_2_ bandgap, crystal structure, defects in it, and NP size [34,35], thus it is not easy to ensure that correct values of refraction index from literature are taken for calculation of *C_A_*. Moreover, the dipole approximation that was satisfied in [27] due to the sufficiently small size of synthesized Ag nanoparticles (<λ_SPR_/10, where λ_SPR_ ≈ 400 nm is silver absorption peak) is not applicable in the present work dealing with NP, which are much larger and do not possess resonant frequency. Thus, in the present paper, model verification by the Beer–Lambert law is adapted when compared to the same method used in [27] to be applicable for the determination of semiconductor NP concentration, and the Mie theory for the calculation of TiO_2_ NP extinction cross-section is exactly applied instead of using the dipole approximation.

## 2. Materials and Methods

A colloidal solution of TiO_2_ NP was synthesized by the pulsed-laser ablation of the Ti target (purity 99.9% and thickness 3 mm, Kurt J. Lesker) immersed in a beaker containing 40 mL of deionized water using Nd:YAG laser (Quantell, Brilliant). Laser specifications are a pulse duration of 4 ns, wavelength of 1064 nm, output energy of 290 mJ and repetition rate of 5 Hz. The laser beam was directed by a system of prisms and focused by a lens (the focal length of 10 cm) onto the target surface. The laser pulse energy in front of the target was 210 mJ while a diameter of a focused pulse on the target surface was 1 mm, which yielded a laser fluence of 27 J/cm^2^. The thickness of a water layer above the target was kept constant at 2 cm during the experiment to keep the ablation efficiency and thus the NP properties, constant [36]. The target was fixed during the experiments to allow the drilling of a crater and thus determine ablated material mass. A scheme of the experimental setup for PLAL is depicted in detail in [37].

After the experiments, the craters created on the targets were studied with an optical microscope (Leica DM2700M, Leica Microsystems, Wetzlar, Germany). Microscopical images were taken at different focal positions with respect to the target surface in order to obtain crater radius dependence on crater depth (i.e., crater profile). Using the obtained crater profile, crater volume is calculated according to the procedure described in [38].

The optical absorbance of laser synthesized colloidal solutions was assessed using a UV-Vis spectrophotometer (Lambda 25, Perkin Elmer, Waltham, MA, USA) in the wavelength range of 200–600 nm.

The crystallinity and crystalline phases were studied by grazing incidence X-ray diffraction (GIXRD). To perform a structural characterization of TiO_2_ NP, the produced colloid was dropped onto a silicon substrate and left to air-dry to obtain TiO_2_ NP film. The crystalline structure of TiO_2_ NP was investigated using a D5000 diffractometer (Siemens, Munich, Germany) in a parallel beam geometry with Cu Kα radiation, a point detector, and a collimator in front of the detector. Grazing incidence X-ray diffraction (GIXRD) scans were acquired with the constant incidence angle α_i_ of 1°, ensuring that the information contained in the collected signal covers the entire film thickness.

The morphology and size distribution of the synthesized nanoparticles was studied by a field emission scanning electron microscope (FE-SEM, JSM-7600F, Jeol Ltd., Tokyo, Japan). Samples for SEM imaging were prepared by depositing one drop of a suspension on a polished Al sample holder. The holder with the specimens was coated with a 3 nm-thick amorphous carbon layer (PECS 682) prior to the SEM imaging. SEM images were analyzed with ImageJ to determine the NP size distribution.

A Zetasizer Ultra (Malvern Panalytical, Malvern, UK) was used to determine the zeta-potential (ζ) by electrophoretic light scattering (ELS). It was calculated from the measured electrophoretic mobility by means of the Henry equation using the Smoluchowski approximation (f(k_a_) = 1.5). Results are reported as an average value of three measurements. The data processing was done by the ZS Xplorer 1.20 (Malvern Panalytical).

Numerical calculations of extinction, scattering, and absorption cross-sections of TiO_2_ NP using equations from Mie scattering theory are performed by Mätzler’s MATLAB code [39].

## 3. Results

### 3.1. TiO_2_ NP Characterization

**Figure 1:**
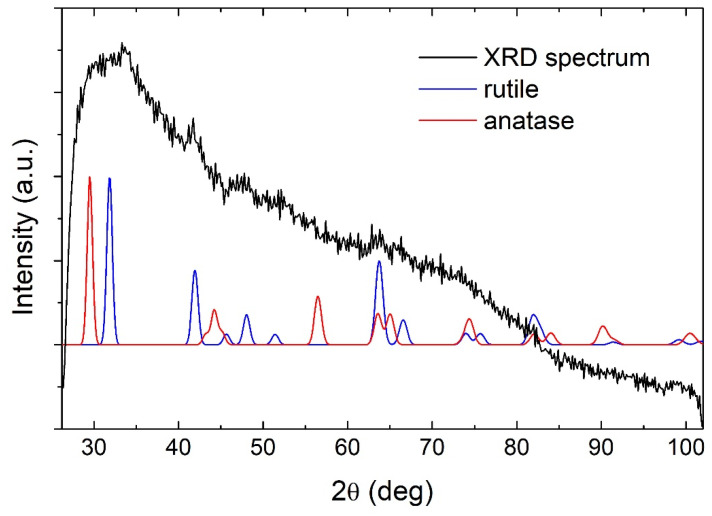
GIXRD pattern of TiO_2_ nanoparticles.

The laser ablation of metallic Ti target in water resulted in the synthesis of a colloidal solution of semiconductor TiO_2_ NP. The GIXRD measurements shown in Figure 1 revealed that TiO_2_ NP are amorphous due to the lack of appearance of any of the major Bragg peaks of TiO_2_ phases (theoretical peaks for rutile and anatase are shown for comparison). The ζ-potential of the produced TiO_2_ NP colloidal solution was measured to be 30 ± 1 mV, making the solution of moderate stability (aggregation and precipitation of nanoparticles appeared after two days). Therefore, TiO_2_ NP concentration can also be considered a homogenous and well-defined value in the as-prepared solution.

**Figure 2:**
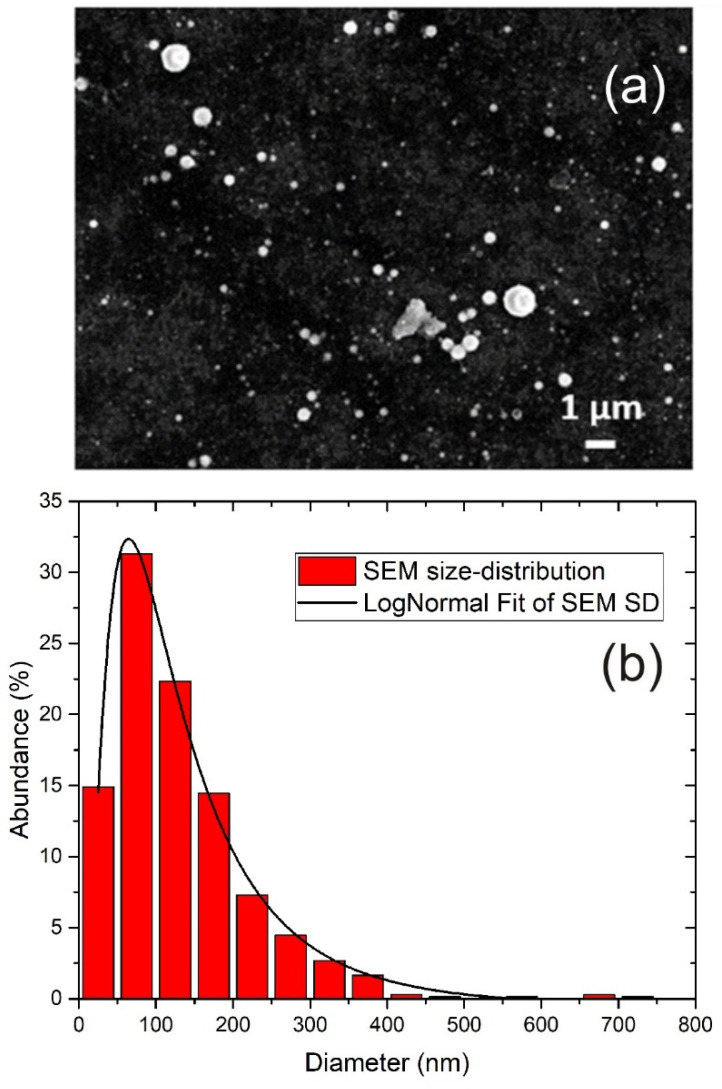
(**a**) SEM image of TiO_2_ nanoparticles (5000 pulses applied in ablation) and (**b**) size distribution of TiO_2_ NP from SEM including Log-Normal Fit.

The morphology and size distribution of TiO_2_ NP were obtained using SEM imaging. A typical SEM image of TiO_2_ NP is shown in Figure 2a, where 5000 pulses were applied in ablation. It can be seen that TiO_2_ NP are spherical and with a broad range of sizes. For each number of laser pulses applied in ablation, the size distribution was obtained by measuring the diameters of 200 single nanoparticles from SEM images. It was found that the size distribution was similar for all tested numbers of pulses. The size distribution obtained from SEM images is shown in Figure 2b.

It can be seen that the size distribution is relatively broad, and it was fitted with the Log-Normal function with the maximum at 64 nm. Log-Normal is a very common function for fitting the size distribution of nanoparticles synthesized by PLAL [40,41,42,43]. Although non-NP species are, to some degree, also present in synthesized colloidal solutions, such as large debris structures, from the obtained size distribution in Figure 2b, it can be assumed that their presence is negligible, so the term “TiO_2_ NP” is justified. Since the nanoparticles are spherical, the average NP volume ${\bar{V}}_{NP}$ is calculated as ${\bar{V}}_{NP}=\frac{1}{6}\bar{d^{3}}\pi$, where *d* is nanoparticle diameter and $\bar{d^{3}}$ can be obtained from the size distribution. From the given size distribution in Figure 2b, the average volume ${\bar{V}}_{NP}$ of nanoparticles is calculated as:

(1) $${\bar{V}}_{NP}=\frac{1}{6}\bar{d^{3}}\pi =\frac{1}{6}\overset{i=M}{\underset{i=1}{\sum }}n_{i}{\left(d_{i}\right)}^{3}\pi$$

where *M* is the total number of size ranges in the size distribution, *d_i_* is the average diameter that corresponds to size range *i* (single column bar in size distribution), and *n_i_* = *N_i_*/*N* is the ratio between the number *N_i_* of nanoparticles corresponding to size range *i* (relative abundance of NP within corresponding column bar) and the total number of nanoparticles *N*. Equation (1) gives ${\bar{V}}_{NP}$ = 3.45 × 10^−3^ μm^3^.

### 3.2. Calculation of TiO_2_ NP Concentration from Crater Volume (C_V_)

The mass of synthesized TiO_2_ NP depends on the amount of ablated material by PLAL, represented as a volume of a crater left on the target after ablation. Due to the fact that the target was made of almost pure titanium (thickness of surface oxidation layer can be neglected when compared to crater volume), the amount of titanium in the formed TiO_2_ NP is coming solely from the target while the oxygen is coming from the water. The volume of the ablated crater *V_crat_* is turned into a fraction of titanium in the synthesized TiO_2_ NP. The total number N of synthesized nanoparticles can be determined from the known volume of ablated material *V_crat_* by performing the following calculation:

(2) $$\begin{array}{c} N\cdot {\bar{V}}_{NP}=\frac{m\left(TiO_{2}\right)}{\rho \left(TiO_{2}\right)}=\frac{m\left(Ti\right)+m\left(O_{2}\right)}{\rho \left(TiO_{2}\right)}=\frac{\rho \left(Ti\right)\cdot Vcrat+m\left(Ti\;\right)\cdot \frac{2\cdot A\left(O\right)}{A\left(Ti\right)}}{\rho \left(TiO_{2}\right)}\\ =\frac{\rho \left(Ti\right)\cdot Vcrat+m\left(Ti\;\right)\cdot \frac{32}{48}}{\rho \left(TiO_{2}\right)}=\frac{5}{3}\cdot \frac{\rho \left(Ti\right)\cdot Vcrat}{\rho \left(TiO_{2}\right)}\; \end{array}$$

where *N* is the total number of nanoparticles in colloidal solution, ${\bar{V}}_{NP}$ is the average volume of nanoparticles, *m*(*TiO_2_*) is the total mass of TiO_2_ NP in colloidal solution, *ρ*(*Ti*) is Ti target density (4.506 g/cm^3^ near R.T.), *ρ*(*TiO_2_*) is the TiO_2_ NP density (3.8 ± 0.1 g/cm^3^) for amorphous TiO_2_ [44], while *m*(*Ti*) and *m*(*O_2_*) are the total masses of titanium and oxygen atoms in TiO_2_ NP in colloidal solution, respectively. *A*(*O*) and *A*(*Ti*) are atomic mass numbers for oxygen atom and titanium atom, which are 16 and 48, respectively. As shown in [45], TiO_2_ NP of 21 nm in size have only 4% larger density than TiO_2_ particles 200 nm in size, so it can be concluded that, due to the prevalence of large nanoparticles in the TiO_2_ NP size distribution (Figure 2b), it is justified to neglect the NP size dependence of *ρ*(*TiO_2_*) in Equation (2).

Number concentration *C_V_* [mL^−1^] of TiO_2_ nanoparticles is then Equation (3):

(3) $$\begin{array}{c} C_{V}=\frac{N}{V_{liquid}}\; \end{array}$$

where total number of synthesized nanoparticles is divided by an amount of liquid *V_liquid_* where synthesis was performed (in our case *V_liquid_* = 40 mL).

**Figure 3:**
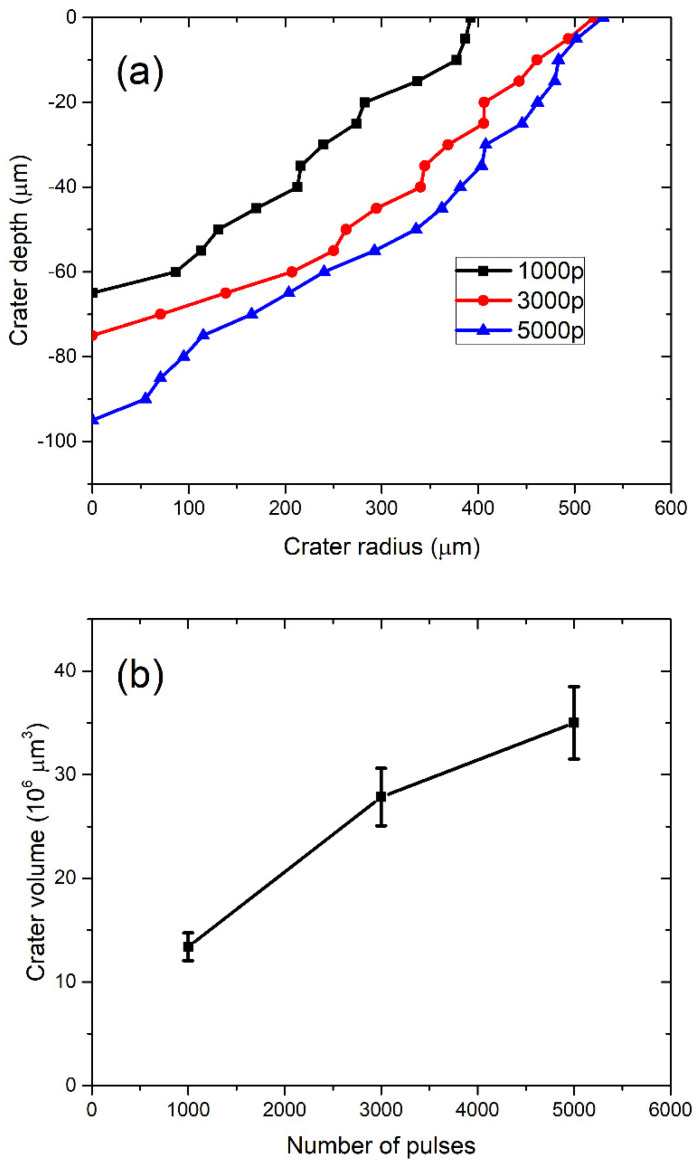
(**a**) Profiles of craters remained on the Ti target after ablation and (**b**) ablation crater volume dependence on the number of pulses.

In Figure 3a, the profiles of craters left after ablation with 1000, 3000, and 5000 applied laser pulses are shown (note that the x–y axis is not in scale). The dashed line denotes the target surface. Craters are characterized by a relatively large aspect ratio (radius to depth), as the depth of a crater is about five times lower than the crater radius. In Figure 3b, the volumes of crater *V_crat_* dependent on the number of pulses are shown, where volumes are calculated from profiles in Figure 3a, as described in [38]. For each number of applied pulses, the total number of nanoparticles in water is calculated by inserting the measured *V_crat_* in Equation (2). It provides the following number of nanoparticles dependence on number of pulses: *N* (1000p) = 7.69 × 10^9^, *N* (3000p) = 15.94 × 10^9^, *N* (5000p) = 20.04 × 10^9^. When the number of nanoparticles *N* is divided by water volume *V_liquid_* = 40 mL, as in Equation (3), the concentration *C_V_* of TiO_2_ NP is obtained for each number of applied pulses. Their values are *C_V_* (1000p) = (1.9 ± 0.2) × 10^8^ mL^−1^, *C_V_* (3000p) = (4.0 ± 0.4) × 10^8^ mL^−1^ and *C_V_* (5000p) = (5.0 ± 0.5) × 10^8^ mL^−1^.

### 3.3. Calculation of TiO_2_ NP Concentration from Beer–Lambert Law (C_A_)

**Figure 4:**
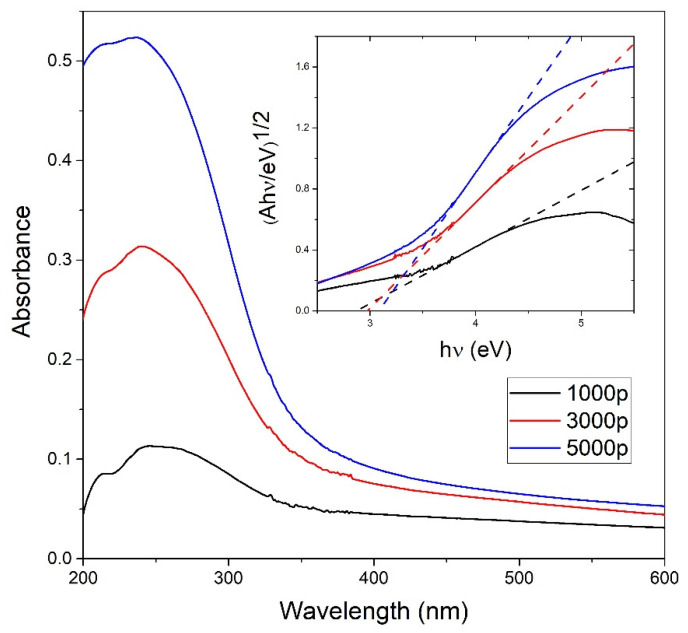
Photoabsorbance vs. wavelength measurements on TiO_2_ colloidal solutions dependent on the number of pulses; Inset: indirect TiO_2_ bandgap calculation from the Tauc plot for different colloidal TiO_2_ NP solutions using the photoabsorbance data.

UV-Vis measurements shown in Figure 4 are performed to obtain the concentration *C_A_* of TiO_2_ NP in colloidal solution using the Beer–Lambert law. Indirect bandgap energies for each TiO_2_ colloidal solution are calculated from the photoabsorbance measurements using the Tauc plot as shown in the inset of Figure 4, and their values are 2.87 eV, 2.98 eV, and 3.08 eV for 1000, 3000, and 5000 laser pulses, respectively. The calculated indirect bandgap energies are close to the indirect bandgap energy 3.0 eV obtained in [46] for amorphous TiO_2_ thin films but are also close to the bandgap energies expected for the most common TiO_2_ phases—rutile (3.0 eV) and to a lesser extent, anatase (3.20 eV) [28].

For the calculation of the NP concentration *C_A_* from the Beer–Lambert law, the same size-distribution data (Figure 2b) was used for *C_V_* calculation. If *ϕ_in_* *(λ)* is the entrant flux, *ϕ_out_* *(λ)* is the output flux of light with wavelength *λ* through the TiO_2_ colloidal solution, *A (λ)* is the absorbance at wavelength *λ*, defined as in Equation (4):

(4) $$A\left(\lambda \right)=-log\frac{\phi_{out\;}\left(\lambda \right)}{\phi_{in\;}\left(\lambda \right)}$$

If it is assumed that the colloidal solution contains spherical TiO_2_ NP within *M* different size ranges, each with a concentration *c_i_* (*i* = 1 to *M*) and average optical extinction cross-section *σ_i_ (λ)* within the size-range *i*, the absorbance *A* can be expressed by the Beer–Lambert law as in Equation (5):

(5) $$A\left(\lambda \right)=\frac{1}{\ln\left(10\right)}\overset{i=M}{\underset{i=1}{\sum }}\sigma_{i}\left(\lambda \right)c_{i}l$$

where *l* = 1 cm is the absorption path length in the cuvette. In our experiment, the absorbance measurements were done immediately after the production of a colloidal solution; as such, there was no precipitation and agglomeration of TiO_2_ NP. From the ζ-potential measurements, it was previously concluded that the TiO_2_ NP colloidal solution was moderately stable. Therefore, the concentration was approximatively the same in the whole solution and can be expressed as:

(6) $$C_{A}=\overset{M}{\underset{i=1}{\sum }}c_{i},\;\;\;\;\;\;c_{i}=n_{i}\cdot C_{A}$$

where *n_i_* is abundance ($\sum_{i=1}^{M}n_{i}=1$) of TiO_2_ NP in the corresponding size range. Abundance *n_i_* is calculated from the size distribution (Figure 2b) for each size range. If the expression in Equation (6) for *c_i_* is inserted in Equation (5), the following expression for concentration *C_A_* is obtained:

(7) $$C_{A}=\frac{A\left(\lambda \right)\cdot \ln\left(10\right)}{l\cdot \sum_{i=1}^{i=M}n_{i}\sigma_{i}\left(\lambda \right)}=\frac{A\left(\lambda \right)\cdot \ln\left(10\right)}{l\cdot {\bar{\sigma }}_{ext}(\lambda )}$$

where ${\bar{\sigma }}_{ext}(\lambda )=\overset{i=M}{\underset{i=1}{\sum }}n_{i}\sigma_{i}\left(\lambda \right)$ is the average extinction cross-section of TiO_2_ NP at wavelength *λ*. *σ_i_* (*λ*) is approximated with extinction cross-section *σ_ext_* *(d_i_, λ)* corresponding to NP diameter d_i_, which is the arithmetic mean of size-range i. The Mie theory is used for the calculation of *σ_ext_* *(d_i_, λ)*. According to the Mie theory, the extinction cross-section *σ_ext_* (*d*) of a spherical particle with diameter *d* is the sum of the corresponding absorption and scattering cross-section, as shown in Equation (8):

(8) σextd=σabsd+σscattd=Qextd·d22π=(Qabsd+Qscattd)·d22π 

where *Q_ext_* is the extinction coefficient, which is the sum of the absorption coefficient *Q_abs_* and the scattering coefficient *Q_scatt_*.

According to [47] *Q_ext_*, *Q_scatt_*, *Q_abs_* can be calculated from Equations (9)–(11):

(9) $$Q_{ext}=\frac{2}{x^{2}}\overset{\infty }{\underset{n=1}{\sum }}\left(2n+1\right)Re\left(a_{n}+b_{n}\right)\;$$

(10) $$Q_{scatt}=\frac{2}{x^{2}}\sum_{n=1}^{\infty }\left(2n+1\right)\left({\left|a_{n}\right|}^{2}+{\left|b_{n}\right|}^{2}\right)$$

(11) $$Q_{abs}=Q_{ext}-Q_{scatt}$$

where *x* is the size parameter defined as:

(12) x=k·d2=2πλnmedium·d2

*k* is the wavenumber of light in a medium (water). *n_medium_* is a real number, due to assumed water transparency. Wavelength-dependent values for water refraction indices used in all calculations are comprehensively listed in [48,49] for water temperature 25 °C. The Mie coefficients *a_n_* and *b_n_* can be calculated from Equations (13) and (14) [47]:

(13) $$a_{n}=\frac{m^{2}j_{n}\left(mx\right){\left[xj_{n}\left(x\right)\right]}^{\prime }-j_{n}\left(x\right){\left[mxj_{n}\left(mx\right)\right]}^{\prime }}{m^{2}j_{n}\left(mx\right){\left[xh_{n}^{\left(1\right)}\left(x\right)\right]}^{\prime }-h_{n}^{\left(1\right)}\left(x\right){\left[mxj_{n}\left(mx\right)\right]}^{\prime }}$$

(14) $$b_{n}=\frac{j_{n}\left(mx\right){\left[xj_{n}\left(x\right)\right]}^{\prime }-j_{n}\left(x\right){\left[mxj_{n}\left(mx\right)\right]}^{\prime }}{j_{n}\left(mx\right){\left[xh_{n}^{\left(1\right)}\left(x\right)\right]}^{\prime }-h_{n}^{\left(1\right)}\left(x\right){\left[mxj_{n}\left(mx\right)\right]}^{\prime }}$$

where *j_n_* and *h_n_* are spherical Bessel’s functions of order *n*. *m* is defined in Equation (15):

(15) $$m=\frac{n}{n_{medium}}$$

where *n* is the TiO_2_ refractive index, which is a complex number, defined as in Equation (16):

(16) $$n=n^{\prime }+i\cdot n^{\prime \prime }$$

where *n*′ and *n*″ are real and imaginary parts of the refractive index, respectively.

In order to calculate wavelength dependence of TiO_2_ NP average extinction cross-section ${\bar{\sigma }}_{ext}(\lambda )=\overset{i=M}{\underset{i=1}{\sum }}n_{i}\sigma_{ext}\left(d_{i},\lambda \right)$ needed for *C_A_* calculation by Equation (7), *n_i_* values were taken from NP size distribution (Figure 2b). Equations (8)–(16) were used for the calculation of $\sigma_{ext}\left(d_{i},\lambda \right)$ for each *d_i_*, with input values of wavelength and wavelength-dependent TiO_2_ and H_2_O refractive indices. The refractive index of amorphous TiO_2_ is similar to the one of anatase, and rutile to a lesser extent, as obtained in [50]. Therefore, using the values of refractive indices reported in the literature for crystallized TiO_2_ is justified for the purpose of estimating the extinction cross-section ${\bar{\sigma }}_{ext}(\lambda )$ of the TiO_2_ NP synthesized in this work. 

To make the best estimation of ${\bar{\sigma }}_{ext}(\lambda )$, calculations were made for three different dependencies of TiO_2_ refractive index on wavelength found in the each of the following works: Siefke et al. [51], Sarkar et al. [52], and Bodurov et al. [53,54], which are comprehensively listed on the website [55]. Siefke et al. [51] performed the WGP (wire grid polarizer) technique for the determination of refractive indices in ALD-prepared TiO_2_ thin film with a thickness of 350 nm in the wavelength range of 120 nm–125 μm using TiO_2_ material with an indirect bandgap at 3.2 eV. Sarkar et al. [52] have used opto-plasmonic sensors for the determination of the refractive indices in TiO_2_ rutile thin film with a thickness of 200 nm in a wavelength range of 300 nm–1.69 μm. Bodurov et al. [53,54] used Bruggeman’s effective medium approximation for the calculation of refractive indices in TiO_2_ anatase nanoparticles (diameter smaller than 35 nm) dispersed in water in the wavelength range 405–635 nm; the measurements were done with a laser micro-refractometer. The calculations of Equations (8)–(16) for the determination of $\sigma_{ext}\left(d_{i},\lambda \right)$ were performed numerically, using Matzler’s MATLAB code [39].

**Figure 5:**
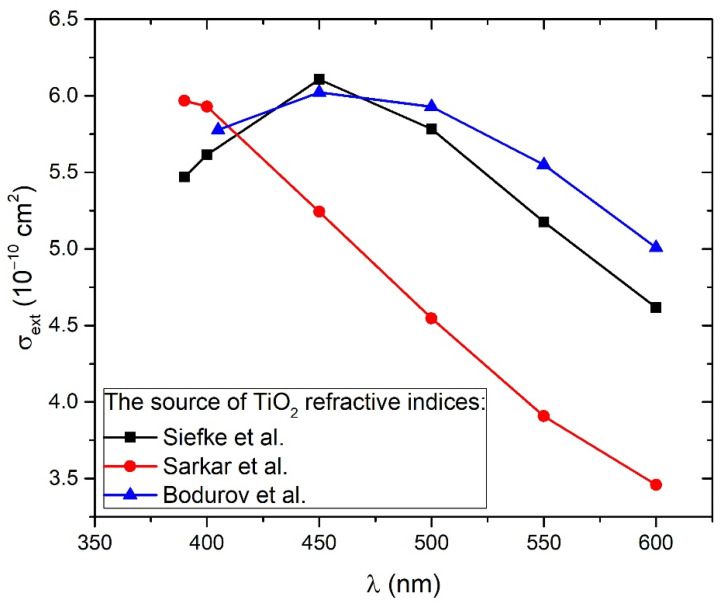
Extinction cross-section dependence on wavelength calculated from the Mie theory for TiO_2_ solution of spherical nanoparticles using SEM size-distribution and TiO_2_ refractive indices from three different works: Siefke et al. [51], Sarkar et al. [52], and Bodurov et al. [53,54] (listed on the website [55]).

Figure 5 shows the wavelength-dependence of cross-sections ${\bar{\sigma }}_{ext}(\lambda )$ calculated for TiO_2_ refractive indices from all of the three mentioned works (Siefke et al. [51], Sarkar et al. [52], Bodurov et al. [53,54]) in the wavelength range 390–600 nm. From Figure 5, it can be concluded that extinction cross-sections for all three cases are very similar to each other at wavelengths 390–415 nm, corresponding to a common TiO_2_ bandgap energy range 3.0–3.2 eV. For larger wavelengths, the difference between them is much greater. Therefore, Equation (7) will provide the best estimations of TiO_2_ NP concentration *C_A_* while inserting *A*(*λ*) and ${\bar{\sigma }}_{ext}(\lambda )$ at wavelength *λ* that corresponds to bandgap energy. Furthermore, both the experimental absorbance *A*(*λ*) and Mie extinction cross-section ${\bar{\sigma }}_{ext}(\lambda )$ have larger values and therefore lower relative error at lower wavelengths, so this is another reason why *C_A_* calculation by Equation (7) has the highest accuracy at wavelengths corresponding to bandgap energy. The third advantage of such an approach is the fact that bandgap energy is easily calculated by the Tauc plot (as in Inset of Figure 4), so it is exactly known which experimental absorbances correspond to the bandgap and should be inserted in Equation (7) for the calculation of *C_A_*. These absorbances are 0.042 for 1000p, 0.072 for 3000p, and 0.090 for 5000p.

**Figure 6:**
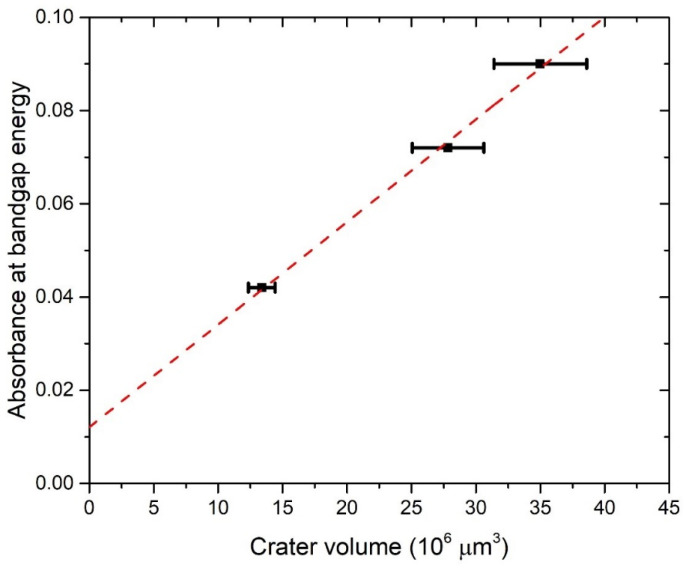
The absorbance at the wavelength corresponding to the bandgap energy vs. the crater volume for TiO_2_ colloidal solution synthesized with PLAL. Linear fit.

Figure 6 shows the dependence of absorbance at bandgap energy on the crater volumes. It can be seen that this dependence is linear, so it means that the average optical cross-section of TiO_2_ NP does not depend on the number of laser pulses. This result is expected due to the similarity in the TiO_2_ NP size distribution at a different number of pulses. Therefore, for each number of pulses, the same average optical cross-section can be inserted in Equation (7) for *C_A_* calculation. The extinction cross-section inserted in Equation (7) for the calculation of *C_A_* is taken from calculations made by using TiO_2_ refractive indices from Siefke et al. [51] and has a value of ${\bar{\sigma }}_{ext}$ = 5.42 × 10^−10^ cm^2^ at wavelength 390 nm (Figure 5), which corresponds to the bandgap of TiO_2_ used in the same paper. The selection of the paper by Siefke et al. [51] is made due to the well-defined TiO_2_ bandgap, which is indirect—like the TiO_2_ NP in this paper. However, the difference in *C_A_* that would occur in the case of using TiO_2_ refractive indices from the other two papers (Sarkar et al. [52], Bodurov et al. [53,54]) is included as the contribution to the uncertainty of *C_A_*. The calculated values of concentrations *C_A_* are: *C_A_* (1000p) = (1.8 ± 0.3) × 10^8^ mL^−1^, *C_A_* (3000p) = (3.1 ± 0.5) × 10^8^ mL^−1^ and *C_A_* (5000p) = (3.8 ± 0.6) × 10^8^ mL^−1^.

## 4. Discussion

**Figure 7:**
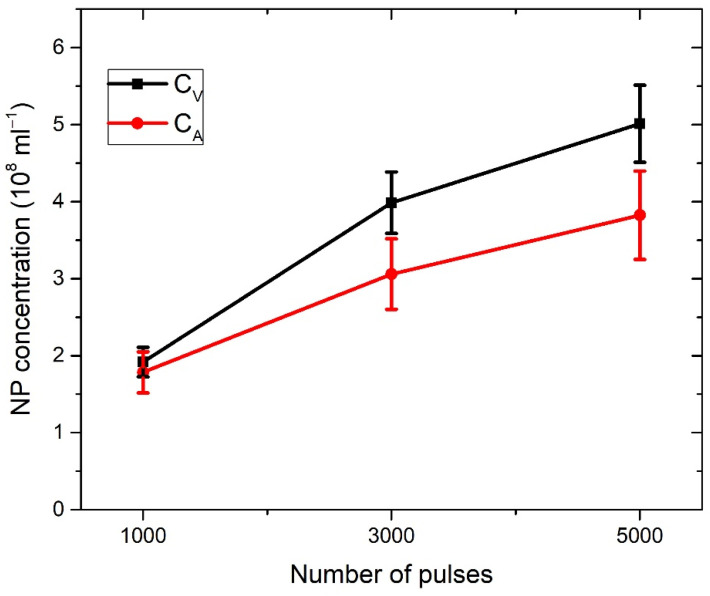
Dependence of TiO_2_ concentration on a number of pulses for two different ways of concentration calculation using crater volume (*C_V_*) or the Beer–Lamber law (*C_A_*).

The concentrations of *C_V_* and *C_A_* as calculated here are both listed in Table 1 and shown in Figure 7 for each number of applied pulses. *C_A_* is close to *C_V_*, but slightly lower: 5% for 1000p, 22% for 3000p, and 24% for 5000p. Therefore, our proposed method for the determination of laser-synthesized TiO_2_ NP concentration from the size distribution and volume of the crater remained on an ablated target is verified, at least within the limits of obtained uncertainty, which are acceptable for many practical purposes. 

There are a few possible explanations why *C_A_* is lower than *C_V_*. First, the actual value of the optical cross-section may be smaller than the calculated value due to the multi-scattering effects that occur on TiO_2_ NP in colloidal solution. Second, there is a possibility that the crater volume was slightly larger than the volume of Ti material included in the formation of TiO_2_ NP. This was due to the crater expansion that may have occurred during the Coulomb explosion in the process of laser ablation or due to the synthesis of structures that are not observable by SEM images, like Ti ions. Third, the TiO_2_ NP analyzed in this paper are amorphous, so they probably have, according to [50], a slightly smaller refractive index than the one used for the calculation of *σ_ext_* from the paper by Siefke et al. [51]. The greatest contribution to the calculated concentration uncertainties is related to NP size-distribution uncertainty, which affects the calculation accuracy of the average volume and average optical cross-section of colloidal TiO_2_ NP.

The proposed method for the calculation of TiO_2_ NP concentration using crater volumes has significant advantages over the method using Beer–Lambert law. In the calculation of concentration using the Beer–Lambert law, a complex Mie theory is applied and, therefore, numerical calculations are needed, the NP refractive indices must be known, there usually exists the upper limit of solution density for the correct absorbance determination, the high concentration homogeneity in solution is required, and in order to have more accurate results, the angular scattering in Mie theory should be considered. These issues do not exist in the method proposed in this article for the calculation of TiO_2_ NP concentration from ablated crater volumes.

## 5. Conclusions

The method for the calculation of PLAL-synthesized NP concentration, originally introduced in our previous work [27] where it is applied for the calculation of pure metallic NP concentration (Ag NP), was herein adapted for the calculation of metal oxide NP concentration (TiO_2_ NP). It is based solely on the determination of the volume of an ablated material crater and the determination of the size distribution of nanoparticles. In order to verify this method, concentrations of TiO_2_ NP in PLAL-synthesized colloidal solutions obtained by the presented method are compared to the concentrations calculated using the Beer–Lambert law. Concentrations obtained from both methods are similar: *C_V_* is 5–25% smaller than *C_A_* in the TiO_2_ NP concentration range (1.8–5.0) × 10^8^ mL^−1^. Therefore, it can be concluded that the proposed method is verified. This method has many advantages compared to traditional methods of concentration determination because it does not require a calibration solution, there is no uncertainty related to the nature of light interaction with nanoparticles, and the only requirement for NP size is the possibility of their detection by microscope. However, to apply this method correctly, the size distribution should be determined with high certainty because it is an important parameter that has a large impact on the calculated value of NP concentration, so this is the main limitation of this method. Furthermore, the method is not applicable when nanoparticles in colloidal solution are very inhomogeneous in terms of their stoichiometry or shape. It can be expected this method will also work for the calculation of the concentration of other metal oxide NP synthesized by PLAL and also while using other types of laser in PLAL, such as a pulsed femtosecond or microsecond laser, but further research may be carried out for confirmation.

## Figures and Tables

**Table 1:** TiO_2_ concentrations calculated from the crater volume (*C_V_*) or from the Beer–Lambert law (*C_A_*) per number of pulses.

No. of Laser Pulses	*C_V_* (10^8^ mL^−1^)	*C_A_* (10^8^ mL^−1^)
1000	1.9 ± 0.2	1.8 ± 0.3
3000	4.0 ± 0.4	3.1 ± 0.5
5000	5.0 ± 0.5	3.8 ± 0.6

## Data Availability

Data included in the paper.
